# SNPdetector: A Software Tool for Sensitive and Accurate SNP Detection

**DOI:** 10.1371/journal.pcbi.0010053

**Published:** 2005-10-28

**Authors:** Jinghui Zhang, David A Wheeler, Imtiaz Yakub, Sharon Wei, Raman Sood, William Rowe, Paul P Liu, Richard A Gibbs, Kenneth H Buetow

**Affiliations:** 1 Laboratory of Population Genetics, National Cancer Institute, National Institutes of Health, Bethesda, Maryland, United States of America; 2 Human Genome Sequencing Center and Department of Molecular and Human Genetics, Baylor College of Medicine, Houston, Texas, United States of America; 3 Genetics and Molecular Biology Branch, National Human Genome Research Institute, National Institutes of Health, Bethesda, Maryland, United States of America; Boston College, United States of America

## Abstract

Identification of single nucleotide polymorphisms (SNPs) and mutations is important for the discovery of genetic predisposition to complex diseases. PCR resequencing is the method of choice for de novo SNP discovery. However, manual curation of putative SNPs has been a major bottleneck in the application of this method to high-throughput screening. Therefore it is critical to develop a more sensitive and accurate computational method for automated SNP detection. We developed a software tool, SNPdetector, for automated identification of SNPs and mutations in fluorescence-based resequencing reads. SNPdetector was designed to model the process of human visual inspection and has a very low false positive and false negative rate. We demonstrate the superior performance of SNPdetector in SNP and mutation analysis by comparing its results with those derived by human inspection, PolyPhred (a popular SNP detection tool), and independent genotype assays in three large-scale investigations. The first study identified and validated inter- and intra-subspecies variations in 4,650 traces of 25 inbred mouse strains that belong to either the *Mus musculus* species or the *M. spretus* species. Unexpected heterozgyosity in CAST/Ei strain was observed in two out of 1,167 mouse SNPs. The second study identified 11,241 candidate SNPs in five ENCODE regions of the human genome covering 2.5 Mb of genomic sequence. Approximately 50% of the candidate SNPs were selected for experimental genotyping; the validation rate exceeded 95%. The third study detected ENU-induced mutations (at 0.04% allele frequency) in 64,896 traces of 1,236 zebra fish. Our analysis of three large and diverse test datasets demonstrated that SNPdetector is an effective tool for genome-scale research and for large-sample clinical studies. SNPdetector runs on Unix/Linux platform and is available publicly (http://lpg.nci.nih.gov).

## Synopsis

Single nucleotide polymorphisms (SNPs) are an abundant and important class of heritable genetic variations, and many of them contribute to genetic diseases. Accurate and automated detection of SNPs as heterozygous alleles in fluorescence-based sequencing traces from diploid DNA samples is challenging because of the low signal-to-noise ratio in the data, and because of sequencing artifacts associated with the various DNA sequencing chemistries.

The authors of this publication have developed a new computer program, SNPdetector, that improves upon existing software tools. The main design principle of SNPdetector was to model the process of human visual inspection of experienced analysts. The new tool is able to cut down significantly on both false positive and false negative discovery rates. Good performance can be achieved, without the need for retraining, in substantially different datasets such as SNP discovery in human resequencing data, mutation discovery in zebra fish candidate genes, and discovery of inter- and intra-subspecies variations in inbred mouse strains. The results demonstrate that this software tool is suitable for the automation of SNP discovery in diploid sequencing traces, and permits a substantial reduction of costly and laborious visual data analysis.

## Introduction

Identification of genetic variations and mutations is important for the discovery of genetic predisposition to complex diseases. Although a wide variety of methods are available for de novo single nucleotide polymorphism (SNP) discovery [1], DNA sequencing is the method of choice for high-throughput screening studies. DNA sequencing may follow either a random shotgun strategy [2–5] or a directed strategy using PCR amplification of specific target regions of interest [6]. As the high-density haplotype map of the human genome [7] nears completion, the demand for large-scale SNP surveys seeking genetic mutations linked to or causative of a wide variety of human diseases (such as diabetes, heart disease, and cancer) is expected to greatly increase [8].

Direct sequencing of PCR-amplified genomic fragments from diploid samples results in mixed sequencing templates. Therefore, one of the most challenging issues in SNP discovery by this method is to distinguish bona fide heterozygous allelic variations from sequencing artifacts, which can give rise to two overlapping fluorescence peaks similar to true heterozygotes. Currently, PolyPhred [9] is the most widely used SNP discovery software for such an analysis. It reports a heterozygous allele only when the site shows a decrease of about 50% in peak height compared to the average height for homozygous individuals. However, inspection of the computational results by human analysts is often required to ensure a low false positive rate, a labor-intensive process.

To provide a sensitive and accurate method for SNP detection in fluorescence-based resequencing, we developed a new software tool, SNPdetector, aiming to “computerize” the manual review process. We report SNPdetector's application in three large-scale genetic variation studies and compare its results with those obtained by human inspection, by PolyPhred, and by experimental validation. In the first study, resequencing was used to validate mouse SNPs discovered by whole-genome shotgun sequencing. The second study identifies novel SNPs in the ENCODE regions of the human genome [10], and the third study aims to discover mutations induced by ENU in 1,236 zebra fish.

## Results

### System Design of SNPdetector

**Figure 1:**
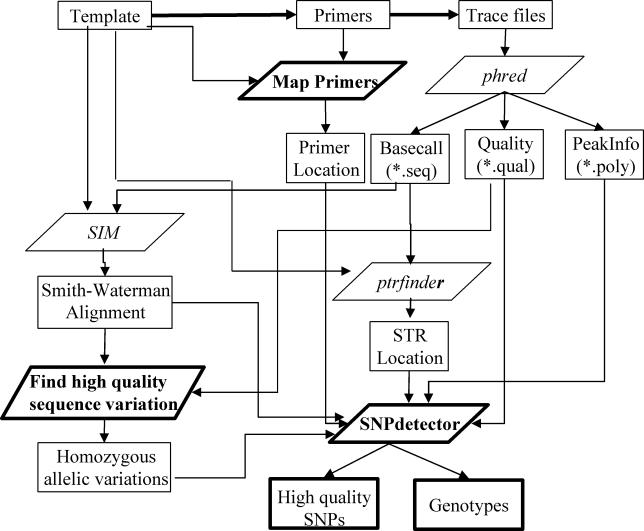
Schematic Diagram of the Principal Steps in the Analysis of Sequencing Variants Found by SNPdetector Paralellograms are analytical modules (usually C programs), and rectangles are input and output data. Programs obtained from the public domain are displayed in italics while those developed in this work are shown in bold. SNPdetector requires the following three sets of input data: (1) a template sequence file, (2) the forward and the reverse sequencing primers, and (3) the trace files. The output includes a list of high-quality SNPs and their genotype calls in each subject.

SNPdetector processes one PCR amplicon at a time with the following four main steps (Figure 1). (1) Run the program Phred [11] to derive base calls, quality scores, and primary and secondary peak information for each trace file. (2) Align sequence reads obtained by resequencing to a reference sequence using SIM [12], a program that implements the Smith–Waterman algorithm. This ensures that all PCR reads are optimally aligned even when there is substantial sequence variation. The ends of the alignments are trimmed, and the user can choose to filter low-quality reads and/or high-quality reads with poor alignments (usually a signal of misassembly). (3) Identify high-quality sequence variations using neighborhood quality standard (NQS) [3], which requires a variation site and each base in its flanking window to exceed a user-defined quality score threshold. NQS was originally developed for automated SNP identification in haploid samples, which is similar to finding SNPs in diploid samples that have homozygous minor alleles. (4) Identify heterozygous genotypes and evaluate the validity of all SNPs. This last step determines the genotype for each sample by analyzing trace files of both forward and reverse orientations. It screens potential systematic sequencing errors by “computerizing” techniques such as horizontal and vertical scanning employed by experienced SNP inspectors. The implementation details are described in Materials and Methods.

During the development of SNPdetector, the mouse resequencing data and a subset of the zebra fish resequencing data were used as training data for developing filters for false positive calls and to determine the lower bound of the signal thresholds for identifying true positive variation. The human resequencing data in the ENCODE regions were not included in training but were used as an independent testing dataset to evaluate the accuracy of the software after training had taken place. The results of the three studies presented here were obtained using the same software configuration.

### Validation and Detection of SNPs in Inbred Mouse Strains

In this investigation we attempted to validate 151 mouse SNPs on Chromosome 16 that were originally discovered by shotgun sequencing of seven laboratory inbred strains [13,14]. We designed 93 sets of forward and reverse PCR primers to assay 40 kb of genomic sequence in 25 inbred strains (details in Materials and Methods of [13]).

During the development of SNPdetector, this dataset was used as the primary training data for identifying sequencing artifacts that are likely to produce false positive SNP calls. The mouse data were chosen for the following reasons. (1) Mouse inbred strains are expected to be either completely homozygous or to have an extremely low rate of heterozygosity as a result of their breeding history. This expectation has been confirmed experimentally by intra-strain variation analysis [14]. Therefore, the vast majority of the heterozygous genotype calls are false positives resulting from sequencing artifacts. (2) In a previous study we analyzed the high-resolution haplotype structure as well as the phylogeny of the mouse strains in the resequenced regions [13]. The knowledge of inbred mouse genetic architecture thus acquired provides an additional reference for resolving ambiguities in SNP validity assessment. (3) The 151 SNPs discovered by genomic shotgun sequencing provide independent verification for SNPs discovered by PCR resequencing.

When using the option to include all sequence reads, SNPdetector identified a total of 1,178 SNPs in all 25 strains. For each SNP, the assembled trace data were manually reviewed using the program Consed [15]. Manual inspection found 11 SNPs to be invalid. SNPdetector found all but two of the SNPs originally discovered by genomic shotgun sequencing. Manual inspection revealed that the two missing SNPs reside in regions where sequences “stutter,” one (dbSNP rs4171354) caused by a polynucleotide track and the other (dbSNP rs4139636) by a simple tandem repeat (STR). Thus these two “SNPs” represent sequence variations resulting from slipped strand extension of DNA polymerase in PCR rather than genetic polymorphisms. Excluding low-quality reads, SNPdetector found 1,019 SNPs, 1,009 of which are valid.

We ran the same dataset using the Phred/Phrap/PolyPhred 5.0.2 package and manually reviewed 187 putative SNPs with score ≥ 30 that were found only by PolyPhred. In the alignments produced by SIM and Phrap, gap locations can vary if they reside in polynucleotide repeats or tandem repeats. As a result, SNPdetector and PolyPhred may produce slightly different locations for a substitution variation adjacent to an insertion/deletion (indel) polymorphism. These discrepancies were manually resolved by comparing the alignments of Phrap and SIM. There were 34 additional valid SNPs in the PolyPhred output, which makes a total of 1,201 valid SNPs when combined with the valid SNPs found by SNPdetector.

Using the 1,201 valid SNPs as a benchmark of the total number of valid SNPs in the mouse resequencing data, we analyzed the error rate for SNPdetector and PolyPhred 5.0.2. The results are summarized in Table 1. SNPdetector, with the run time option of including all sequence reads, has the lowest false positive and false negative rates (0.93% and 2.58%, respectively). In the output of PolyPhred, the results obtained from score ≥ 97 have the lowest false positive rate (5.31%) and those from score ≥ 30 have the lowest false negative rate (14.73%).

**Table 1:**
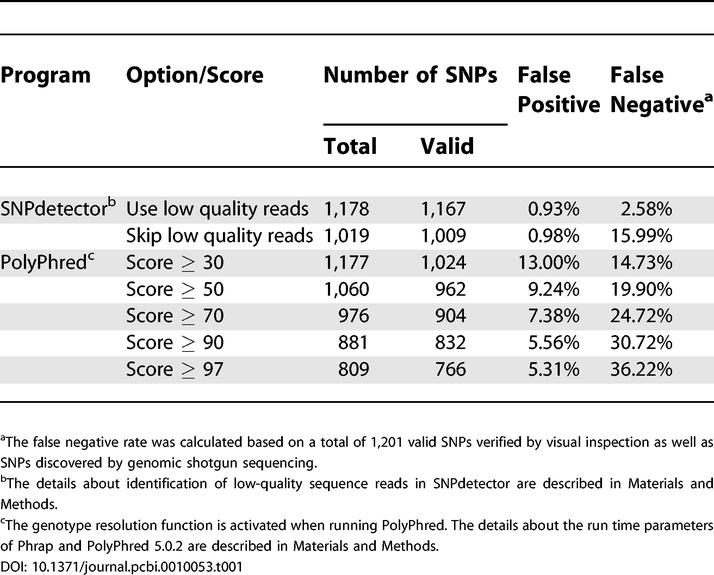
Comparison of the Results Obtained by SNPdetector and PolyPhred (Version 5.0.2) in Mouse Resequencing

### Discovery of Heterozygous Alleles in the Wild-Derived Inbred Mouse Strain CAST/Ei

In the mouse sequencing data described above, SNPdetector identified 11 putative SNPs with heterozygous genotypes in at least one of the 25 inbred strains. As part of the quality assurance process, we manually reviewed all of these SNPs. Six of the putative SNPs were false positives arising from background noise. However, manual inspection detected no sequencing artifact in the remaining five markers. The markers are located at two genomic loci. One of the two loci encodes the *EphA6* gene, and the two wild-derived inbred strains, MOLF/Ei and SPRET/Ei, are heterozygous at three putative SNP sites. The other locus encodes *Bach1,* a heme-binding transcription factor. CAST/Ei, a wild-derived inbred strain of the *Mus. mus. castaneus* subspecies, was heterozygous at two putative SNP sites located at the 3′ UTR of *Bach1*. None of the five markers has homozygous minor alleles.

To determine the validity of the unexpected heterozygosity in the inbred strains, we redesigned sequencing primers to assay DNA samples of the original animals as well as additional animals of CAST/Ei (three animals), MOLF/Ei (one animal), and SPRET/Ei (one animal) strains. The observed heterozygosity at the *EphA6* locus turned out to be an artifact of genomic duplication coupled with polymorphisms of MOLF/Ei and SPRET/Ei strains at the sequencing primer binding sites. However, no genomic duplication was found for the *Bach1* locus using the current mouse assembly (March 2005 release of NCBI build 34). Genotypes of the four CAST/Ei animals are summarized in Table 2. One CAST/Ei animal was homozygous at the first SNP site while all other sites were heterozygous (Figure S1). The minor alleles in the CAST/Ei heterozygote were the same as those represented in the human orthologous sequence.

**Table 2:**
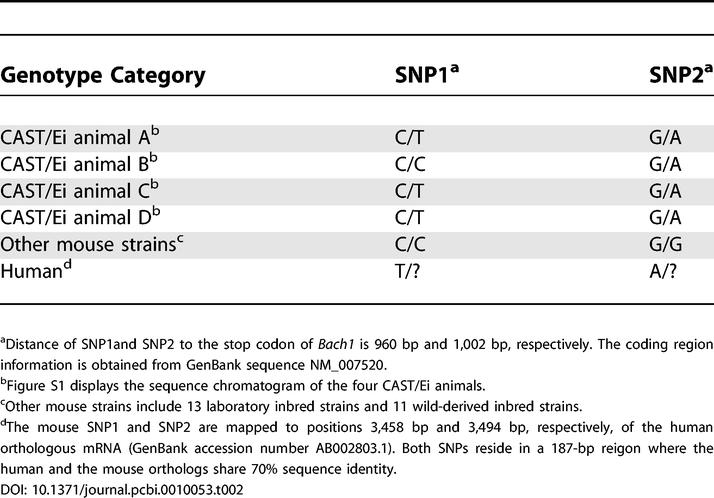
Heterozygosity at the *Bach1* Locus in Animals of Wild-Derived Inbred Strain CAST/Ei

### SNP Discovery in Human

We used SNPdetector for SNP discovery as part of the HapMap project [7]. In the SNP discovery phase 48 unrelated individuals from four populations were chosen for resequencing: 16 from the Centre d'Etude du Polymorphisme Humain collection [16]; 16 from Yoruba individuals from Ibadan, Nigeria; eight from Japanese individuals from Tokoyo, Japan; and eight from Han Chinese individuals from Beijing, China. Cell lines for each are available from the Coriell Institute for Medical Research (http://locus.umdnj.edu/nigms/products/hapmap.html).

A total of 11,241 candidate SNPs were found across all regions, of which approximately half (51.9%) were novel, compared to data in build 121 of dbSNP. In all, 80% of the SNPs had a minor allele frequency greater than 0.05.

Nearly 6,000 of the SNPs identified by SNPdetector were selected for genotyping in expanded assay panels using a variety of commercial and academic genotyping platforms as part of the International HapMap Project. The larger panels consisted of the following samples: 90 from the Centre d'Etude du Polymorphisme Humain collection; 90 from Yoruba individuals from Ibadan, Nigeria; 45 from Han Chinese individuals from Beijing, China; and 45 from Japanese individuals from Tokoyo, Japan; the 16 individuals used in the SNP discovery phase were a subset of the genotype panel. Candidate SNPs that turned out to be monomorphic across all populations were considered to be false positives. The false positive rate ranged from 2% on Chromosome 12 to 5.8% on Chromosome 7 (Table 3). The overall SNP validation rate was 95.5%.

**Table 3:**
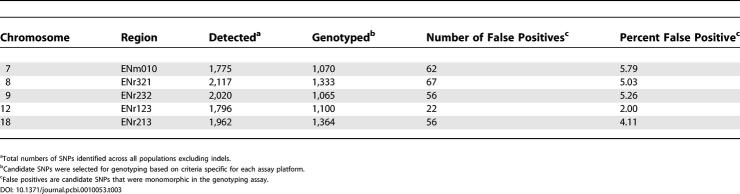
Genotyping of Candidate SNPs Identified by SNPdetector in Human ENCODE Regions

To compare the performance of SNPdetector with the other SNP detection programs, we reanalyzed a subset of ENCODE data (61 amplicons on Chromosome 18) using PolyPhred 5.0.2 and NovoSNP [17] (a new SNP detection software package). We did not run this analysis on the entire ENCODE dataset because for computational SNPs that do not have genotype data, we had to manually review sequence traces to assess their validity.

A total of 85 valid SNPs were found by at least one of the three programs, 71 of which were verified by experimental genotyping, and 14 by manual review alone. The results, summarized in Table 4, show that the false positive and the false negative rates of SNPdetector are much lower than those of the other two programs for this dataset: the combined false positive and false negative error of SNPdetector is approximately half of the lowest error rate in PolyPhred and one tenth of that in NovoSNP.

**Table 4:**
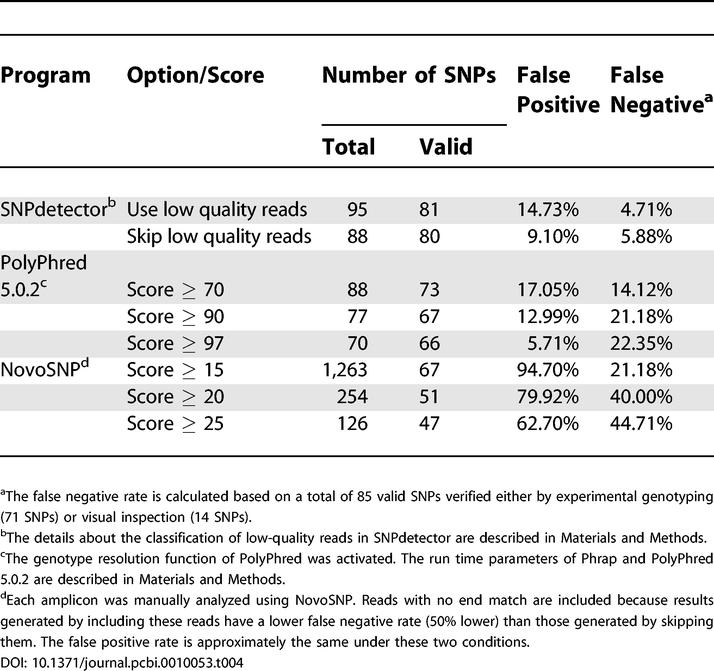
Comparison of SNPdetector with PolyPhred 5.0.2 and NovoSNP on a Subset of ENCODE Data

### Detection of ENU-Induced Mutations in 1,236 Zebra Fish

A total of 26 pairs of forward and reverse primers were designed to identify ENU-induced mutations in 1,236 zebra fish in several candidate genes. The fish population is expected to have common and rare polymorphisms in addition to mutations. Each mutation is expected to be present as a heterozygote in only one fish, resulting in minor allele frequency of 4.0 × 10^−4^ in the overall population.

To detect mutations at such low frequency requires high sensitivity of the computational tool. Therefore, a subset of the zebra fish data was used as the training data for developing modules that distinguish weak signals from sequencing artifact.

We then ran SNPdetector to discover all candidate genetic variations in the entire dataset. Those that had only one heterozygote across the entire population were considered to be putative mutations. These putative mutations were manually reviewed and subjected to repeated sequencing. SNPdetector identified all eight verified mutations. A total of 102 SNPs with minor allele frequencies ranging from 0.2% to 50% were also identified. To find all mutations using PolyPhred 5 requires setting a score threshold of six (the highest PolyPhred score is 99). At such a low threshold, the majority of the variations identified are expected to be false positives.

### Sequence Coverage of the Mouse, Human, and Zebra Fish Datasets

We analyzed the sequence coverage to estimate the overall false negative rate resulting from rejection of low-quality bases by SNPdetector. Sequence coverage refers to the percentage of the total bases that are accepted for SNP identification by the program. In this analysis, each aligned base was subjected to the acceptability test employed by SNPdetector, which evaluates its short-range and long-range quality score distribution as well as secondary peak profile (details in Materials and Methods).

**Figure 2:**
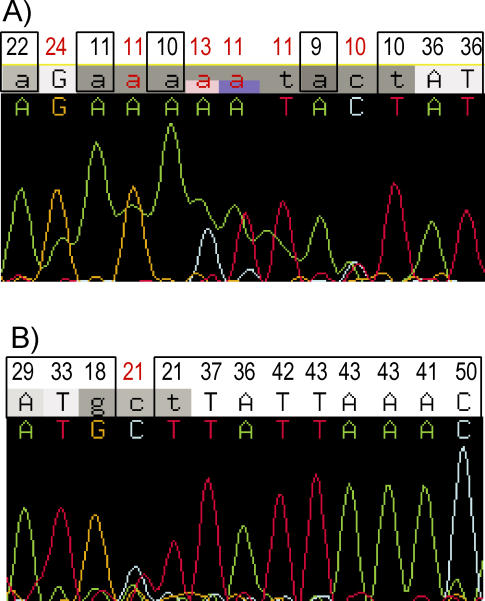
Rejected and Accepted Bases in a Sequence Trace The Phred quality scores are indicated at the top. The quality scores for rejected bases are labeled in red. Accepted bases are marked by rectangular boxes. (A) A subregion of polyA bubble showing that low-quality bases with no secondary peaks are accepted by SNPdetector. (B) A subregion showing that a Q20 base is rejected because of its high secondary peak even though the majority of neighboring bases have high-quality scores.

We calculated the read-based coverage and the sample-based coverage; the latter combines the forward and the reverse reads from the same sample (details in Materials and Methods). The results are summarized in Table 5. In the read-based coverage analysis, 89%–91% of the total bases were accepted; 3%–4% of the total bases were rejected because of stutter, showing that stutter accounts for 30%–40% of all rejections. Q20 bases (e.g., bases with Phred quality score ≥ 20) constituted 89%–90% of the total bases, while their percentage in the accepted bases was higher (in the range 95%–98%), indicating that a good proportion of rejected bases are of low quality. However, quality score alone does not determine the status of a base. In the read in Figure 2, four bases with very low quality scores (in the range of nine to 11) were accepted because they had no secondary peak background (Figure 2A) while one Q20 base was rejected because of its background noise (Figure 2B).

**Table 5:**
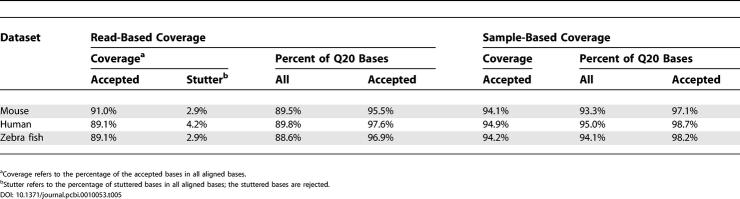
Sequence Coverage Analysis of the Three Datasets

When we combined forward and reverse reads from the same sample to calculate coverage, 94%–95% of the total bases in the three datasets were accepted. The sample-based coverage gives a more accurate estimate of false negative rate resulting from lack of coverage than the read-based coverage because (1) in all three datasets each sample was sequenced in both orientations; (2) SNPdetector analyzes both the forward and reverse reads from the same sample to obtain the genotype; and (3) sequencing artifacts such as stutter have a complementary pattern in the forward and the reverse reads, e.g., stutters in one orientation usually have non-stutter bases in the opposite orientation.

## Discussion

We have demonstrated the ability of SNPdetector to accurately call SNPs in resequencing reads from PCR templates with very low false negative rates (2%–6%) and acceptable false positive rates (1%–9%). In the test data analyzed here, the error rate of SNPdetector is much lower than that of the two alternative methods: PolyPhred (version 5.0.2) and NovoSNP (see Table 4). InSNP [18] is another recently developed SNP analysis software package. We did not reanalyze the test data using this tool because its main function is to support interactive human inspection rather than perform automated data analysis. The false positive rate of InSNP was reported to be in the range of 93% to 95% [18].

**Figure 3:**
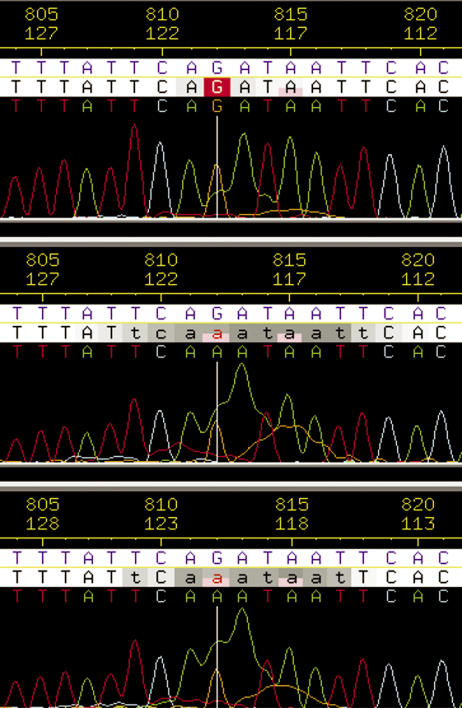
A PolyA Bubble That Occurs in Multiple Samples The bubble was recognized as a sequencing artifact by SNPdetector, and no SNP was called even though the alternative adenine residue (in the highlighted column) appeared in two samples with an average Phred quality score of 20. In addition, all three traces in this region have a polyG spill at the right, with a secondary guanine peak spanning four residues; and a polyT spill at the left, with a secondary thymine peak spanning three residues.

SNPdetector is able to find SNPs or mutations of very low frequency because it does not rely on multiple instances of a minor allele to evaluate SNP validity. In our experience of manual SNP review, we have observed that sequencing artifacts such as stuttering, bubbling, and spilling usually occur in multiple samples at the same locus (Figure 3). In the case of stuttering, the sequence artifact can be attributed to sequence repeat content (i.e., polynucleotides or STRs) or indel polymorphism. Thus, noise that reduces the accuracy of SNP detection can be systematic and highly reproducible. Multiple observations of a genotype are considered confirmatory only if they were derived from sequence reads of the same sample in opposite orientations, because complementary bases are assayed in the forward and reverse sequence reactions.

The sensitivity of SNPdetector enabled us to discover unexpected heterozygosity in the inbred strain CAST/Ei. Of the 1,167 mouse SNPs, two located at the 3′ UTR of *Bach1* were heterozygous in CAST/Ei strain while the remaining 24 strains were homozygous. This discovery was confirmed by repeated sequencing of additional animals of CAST/Ei strain using a different pair of sequencing primers. All but one of the genotypes were heterozygous (see Table 2), suggesting that maintaining heterozygosity at this locus might be critical to CAST/Ei. Though heterozygosity of noncoding DNA was previously shown in recombinant inbred strains [19], this is the first case to our knowledge in which heterozygosity is observed in the mRNA transcript of a well-established inbred strain. Maintenance of heterozygosity is expected to be accompanied by reduced fecundity, and CAST/Ei is known to have smaller litter size than other inbred strains [20].

The three studies presented here include regions of very high SNP density. For example, among the regions with the highest SNP density, one 622-bp zebra fish amplicon contains 11 SNPs and one 854-bp mouse amplicon contains 26 SNPs. The genetic divergence in these regions can lead to the generation of multiple contigs if we attempt to assemble all the sequence reads, and the errors in the alignments of an assembly can become a major source of SNP detection error. In the mouse study, one of the strains is SPRET/Ei. It belongs to *M. spretus,* not *M. musculus;* the other 24 strains belong to the latter species. In the data analyzed here, the variation rate between *M. spretus* and *M. musculus* is approximately one every 50 bp, indicating SNPdetector can be useful for identifying inter-species variations of highly related organisms.

During the development of SNPdetector, we used the mouse resequencing data as the training dataset because heterozygotes in inbred mouse strains are almost always false positive as a result of mouse breeding history. This allowed us to investigate potential sources of false positive heterozygous allele calls and develop filters for these sequencing artifacts. The initial design of SNPdetector had included an option to allow a user to set the threshold on the quality measurement of individual genotype. However, with effective filtering, a low-quality threshold increased the sensitivity but not the false positive rate of SNP detection, making user intervention unnecessary.

In the human resequencing data, the assessment of SNP validity was based on experimental genotyping in those cases where the data were available. We resorted to visual inspection of trace data only when there were no genotyping data (see Table 4). However, we noticed that in some cases genotype data were inconsistent with the result of visual analysis. For example, in the test data presented in Table 4, four visually apparent SNPs were scored as monomorphic in genotyping. These were rare SNPs with one heterozygote in the resequencing population; each SNP had both forward and reverse sequence coverage (Figure S2). On the other hand, two visually rejected SNPs were scored as polymorphic in genotyping; neither was found by SNPdetector or PolyPhred. Taking into account the results of visual analysis, the false positive and false negative rates of SNPdetector would be 4.55% and 3.45%, respectively, much lower values than those in Table 4. Clearly, the error rates of the genotyping assays, though not yet available, must be taken into account when used as a standard for assessing the accuracy of SNP discovery.

The current version of SNPdetector is able to find indel polymorphisms when there are homozygous minor alleles. The stutters caused by heterozygous indels are detected but not decoded, partly because of the difficulty in distinguishing the stutters caused by indel polymorphisms from those caused by polynucleotide runs or STRs. Additionally, the “.poly” files generated by Phred for peak analysis only include primary and secondary peak information. However, the secondary peaks may not always represent one of the two reads downstream of a heterozygous indel if there are sequencing artifacts in the region. We are currently evaluating the possibility of revising Phred to export quality scores of the secondary peaks to facilitate the decoding of heterozygous indels.

The current version of the program does not implement sequence assembly because, with the successful completion of the Human Genome Project and genome projects in other species, high-quality reference genomic sequences are readily available for human and other model organisms. An assembly module could be easily incorporated if SNPdetector were used to analyze an organism lacking a high-quality reference sequence.

At this level of accuracy, the success rate of the resequencing reactions is now the limiting factor in screens for identifying novel SNPs and mutations. SNPdetector runs on Unix and Linux and is publicly available by anonymous ftp (http://lpg.nci.nih.gov).

## Materials and Methods

### Human ENCODE resequencing.

PCR amplicons were designed to tile five human genome ENCODE [16] regions (ENm110 on 7p15.2, ENr321 on 8q24.11, ENr232 on 9q34.11, ENr123 12 q12, and ENr213 on 18 q12.1), each 500 kb in length. In total, 4,190 PCR reactions were carried out on each individual to amplify the 2.5 Mb of genomic sequence.

PCR reactions were run in 6-fold multiplex reactions arranged so that consecutive amplicons were never in the same reaction. To the 5′ end of each primer pair were attached specific sequencing primers so the reaction mixture could be sequenced directly upon completion of the amplification. DNA sequences were tested for fidelity to the intended amplicon sequence by comparison to the human genome using BLAST. Sequencing reads that failed to make their best match to the genome between the sequencing primers were rejected from SNP analysis. In all, 258,909 sequences met this quality criterion and went on to SNPdetector. SNPdetector aligned the DNA sequences to the reference (NCBI build 34 of the human genome), and then the program called SNPs. From this analysis, 11,241 candidate SNPs were identified, including 1,571 homozygous indels. Approximately one-third of the candidate SNPs were not eligible for genotyping because they failed to meet criteria for assay design. These criteria exclude SNPs lying in palindromes, AT- or GC-rich regions, or repeated DNA (see http://www.hapmap.org/downloads/assay-design_protocols.html).

### Programming language and system requirements.

SNPdetector was implemented in C and Perl. It currently runs on Unix and Linux platforms.

### Identification of low-quality or misassembled reads.

Each subject sequence is aligned to the reference sequence; the two ends of each read are trimmed until there is a 20-bp window with 95% or greater identity to the reference sequence at each end. Sequences that lack such a window are not included in SNP detection.

The trimmed alignments are then used to evaluate the read quality. A read is considered poor quality if it does not have a subregion (at least 30 bp) with high sequence quality (Phred quality score ≥ 30 in 90% of the bases) as well as high sequence identity (95%) to the reference sequence. We did not require high average quality across the entire sequence because such measures tend to exclude stuttered reads (caused by STR, polynucleotide, or indel polymorphism). The average quality score for a stuttered read is usually low but the bases upstream of the stutter can be of sufficient quality for SNP analysis. If a sequence has a high quality (defined previously), highly divergent (<70% identity to the reference sequence) subregion, then the read is considered a “misassembled” sequence not suitable for SNP detection.

The parameters were derived from empirical analysis of genomic regions with high SNP density. However, they are adjustable in the pipeline, and the user can opt out of the low-quality/misassembly check.

### Modification of NQS for detecting SNP and indel polymorphisms.

Prior to heterozygote detection, we implemented a modified version of NQS to identify putative SNP and indel polymorphisms that have homozygous minor alleles in the resequencing sample. First, the many-to-one alignments computed by SIM [12] were converted to an *M* × *N* multiple alignment by projecting insertions in subject sequences as deletions in the reference sequence. *M* corresponds to the alignment length; *N* corresponds to the number of subject sequences. A position in *M* is considered a putative variation site if it has more than two qualified alleles across *N* samples.

A residue is considered a qualified substitution allele if it and each base in its 4-bp flanking regions has Phred quality score ≥ 15. This minimum quality score is an adjustable parameter in the program. If a residue fails this standard NQS check but resides in a 9-bp window where the quality score of each base exceeds 25, then it is qualified by this expanded window NQS. The latter criterion ensures that a high-quality residue is included even if there is sequencing artifact on one side of the 4-bp flanking region.

A higher-quality threshold is applied to qualify a putative indel because errors in base calling tend to generate false indel polymorphisms. The minimum quality score of NQS is 25 instead of 15. If a putative indel resides in a polynucleotide repeat, then the entire repeat as well as the 4-bp flanking region of the repeat is required to pass the NQS quality check because in a repeat polymorphism the gap location is arbitrary. The expanded window NQS is not used for indel allele qualification because alignment artifacts resulting from base calling errors often produce false indel alleles.

If a sample has forward and reverse reads and its sequence in the opposite direction is identical in the 10-bp regions flanking either side of the putative allele, the quality scores from the two reads are combined for the NQS check. When there is a discrepancy in the forward and reverse reads of a putative indel allele, the indel allele is disqualified.

Putative substitutions and indels identified by NQS are subjected to further evaluation in the process described below. Those that fail in subsequent test are not listed in the output.

### Ratio of primary to secondary peak in heterozygote detection.

The zebra fish resequencing data had more than 1,200 subjects sequenced in both forward and reverse orientations. The large sample size in this study allowed us to inspect the distribution of secondary-to-primary-peak ratio as noise in “dirty” homozygotes (e.g., homozygotes with a secondary peak background) and as signal in true heterozygotes. We found that it was not uncommon for a homozygote to have a secondary peak approximately 20% of the primary peak height. On the other hand, it was uncommon for a true heterozygote to have a secondary peak less than 30% of the primary peak. Therefore, in the default setting we used the 30% threshold as the lower bound for detecting putative heterozygotes; a secondary peak below 20% of the primary peak was considered background noise in a dirty homozygote in the initial genotype assessment.

### Genotype quality classification.

The genotype of a sequence read is classified into one of the following six categories: high, med, low1, low2, low3, and reject. The quality class is determined by analyzing the Phred quality score of the variant site and its 4-bp flanking side. The threshold of each quality class is based on previous empirical analysis of NQS accuracy [3,4] (e.g., the minimum quality score for each base in the flanking regions is 15) as well as comparison with the validated SNPs in the training datasets. The initial assignment of a genotype quality class may be modified by the subsequent processing described below under “Horizontal and Vertical Scan.”

**Figure 4:**
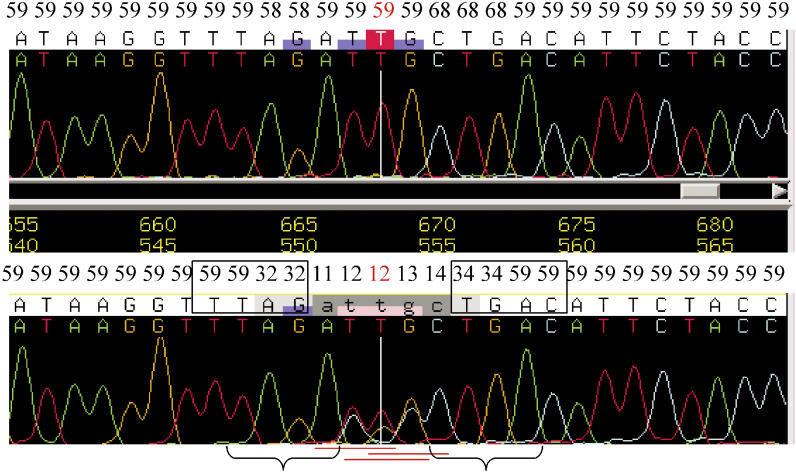
Sequence Traces of a SNP Cluster with Three Consecutive SNPs The top is a homozygous sample and the bottom a heterozygous one. The Phred quality score is labeled on top of each base. In the heterozygous sample, the three HQDPs around the three heterozygotes are labeled with red lines at the bottom. The flanking bases used for calculating genotype quality class of the highlighted heterozygote in the middle are marked by rectangular boxes, which do not include any HQDPs. The flanking bases used to assess background noise in the flanking region are labeled with brackets at the bottom.

In Phred, one of the four parameters for discriminating errors from correct base calls is “uncalled/called ratio,” e.g., the ratio of the height of the largest uncalled peak to the smallest called peak within a 7-bp window around the current site [11]. In many cases, the uncalled secondary peak at a heterozygote site is the largest uncalled peak in this 7-bp window. As a result, the Phred quality scores of a heterozygote and its flanking bases can be much lower than those of a homozygote with a similar peak profile. Such an example is shown in Figure 4. The most dramatic Phred quality score drop is found at the heterozygote site and its immediate 1-bp neighbors. We define these three bases as a “heterozygote Phred quality score drop unit” (HQDU). The flanking region is analyzed under two conditions: (1) using the 4-bp flanking region around the current site without taking into account the HQDUs at the site and within its flanking region, and (2) using only those bases that do not belong to HQDUs at the site and within its flanking region. If there are fewer than four such bases within 20 bp of the site, then the flanking region is considered invalid (score set to zero). The 20-bp constraints ensure that a region that consists entirely of HQDUs (as in the case of stutter) is ignored. The maximum of conditions 1 and 2 is used to represent the flanking region score of the site because condition 1 is more accurate if the maximum uncalled peak (defined by Phred) in the 7-bp window is not the secondary peak of a heterozygote but a sequencing artifact (such as bubble). On the other hand, a score derived from condition 2 is more accurate if a heterozygote is the maximum uncalled peak in the region. An example of skipping HQDUs in the flanking region analysis is shown in Figure 4.

Once the flanking region is defined, the genotype score is calculated as follows. If each base at the flanking region exceeds a Phred quality score of 15, 25, or 40, then the flanking region is assigned a score of one, two, or three, respectively. If the variation site exceeds a Phred quality score of 15, 25, or 40, then it is assigned a score of one, two, or three, respectively. If the average quality score of the flanking region exceeds 25 or 40, then the score is incremented by one or two, respectively. If the average is below 15 (low), then a penalty of −1 is imposed. The combined total score is then used to derive the initial classification group as follows: score < 0 → reject; score = [0,1] → low3; score = 2 → low2; score = [3,4] → low1; score = [5,6] → med; and score ≥ 7 → high. Thus, a genotype of class “reject” has Phred quality score below 15 at the site as well as the flanking region; such a site will not be used for SNP detection.

The initial genotype quality class can be turned into the class reject in horizontal or vertical scan analysis. In these scans, a putative heterozygote may also be reclassified as a dirty homozygote or vice versa. When a genotype changes its status, the quality class is also recalculated.

### Genotype noise assessment.

To evaluate noise within the 4-bp flanking region of a putative heterozygote or homozygote, the program checks the secondary peak of each base in the flanking region. As above, define *p* = (secondary_peak_area/primary_peak_area) × 100 (i.e., percent of primary peak area occupied by secondary peak). If each base in the flanking region passes the test of *p* = 0, *p* ≤ 10, or *p* ≤ 20, then the flanking region is considered to have no, little, or limited noise, respectively. A site with a *p* > 70 secondary peak in the flanking region is skipped to avoid penalizing a putative heterozygote in a SNP cluster (see an example in Figure 4). The same test is applied to measure the noise level at the site of a homozygote. For a putative heterozygote, the higher the *p*-value, the stronger the signal. A classification of no, little, and limited noise is awarded to putative heterozygote sites with *p* ≥ 80, *p* ≥ 50, and *p* ≥ 30, respectively.

### Horizontal and vertical scan.

Manual SNP inspection usually involves a horizontal scan of the same trace and a vertical scan across multiple traces at a putative SNP site. The horizontal scan assesses whether the signal is distinguishable from local noise, while the vertical scan determines whether the signal is distinguishable from the noise in the other samples.

To model the horizontal scan, SNPdetector first identifies short-range (5 bp) and long-range (>50 bp) features indicative of potential problems in a sequence read. These are (1) regions with low sequence similarity, (2) regions with low sequence quality, and (3) regions with a high secondary peak background. Long-range features, when occurring downstream of a STR (computed with the program Ptrfinder [21]) or a polynucleotide track (≥8 bp), or an indel polymorphism identified in the NQS analysis, usually indicate stuttering in PCR amplification [22], and stutters are disqualified (e.g., genotype quality class set to reject). Short-range features accompanied by specific types of flanking sequence indicate potential artifacts—e.g., spilling (i.e., a background of secondary polynucleotide track extended from a neighboring primary peak; see Figure 3), bubbling (i.e., reads embedded underneath a polynucleotide blob; Figure 3), or factitious indels resulting from base-calling errors. Details of the parameters used for horizontal scan are summarized in Table 6.

**Table 6:**
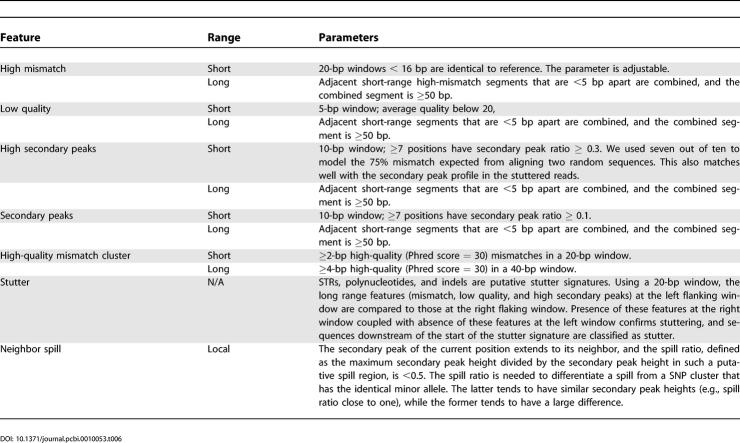
Parameters Used in Horizontal Scan

To model the vertical scan of a human inspector, SNPdetector first identifies high-quality homozygotes with no secondary peak (e.g., secondary-to-primary-peak-area ratio is zero) in the 10-bp flanking region. These “clean” homozygotes are then used to find “dirty” homozygotes. A dirty homozygote is determined to be present if one of the following conditions is true: (1) there is a discrepancy between forward and reverse reads of the same sample, e.g., a clean homozygote is found in sequence read of one orientation while the read in the opposite orientation has a secondary peak; or (2) a sequence read has a low secondary peak (secondary-to-primary-peak-area ratio < 10) and no reduction of its primary peak compared to that of a clean homozygote derived from the same orientation. To adjust for baseline differences in peak area measurements in different traces, we used a relative score, i.e., the ratio of the primary peak area at the putative site to that of its immediate homozygous neighbors. The unclassified traces are processed in ascending order of their secondary-to-primary-peak-area ratio, and each is compared to reads of clean or dirty homozygotes to determine (1) whether its secondary peak area is comparable to the secondary-to-primary-peak-area ratio found in dirty homozygotes, and (2) whether the reduction of its primary peak is comparable to those observed in dirty-to-clean homozygotes. A sequence read deemed indistinguishable from a classified homozygote is considered to represent a dirty homozygote and is included in the analysis of the remaining data. A more stringent threshold is used for reads with short-range features indicative of sequence artifacts identified during the horizontal scan. The genotype quality class and noise class are recomputed for putative heterozygotes reclassified as dirty homozygotes.

The complete list of parameters used for the horizontal and the vertical scan is listed in Table S1.

### Analysis of sequence coverage of the three datasets.

Sequence coverage refers to the percentage of the total bases that are accepted for SNP detection by the software. Empty trace files, reads with unacceptable quality (details under “Identification of Low-Quality or Misassembled Reads”), and reads that fail to make their best match to the reference sequences are considered assay failures. They are not used as an input for SNPdetector and, as a result, are not included in the coverage analysis.

The total number of resequenced bases in a read includes every base that is aligned to the genomic interval spanned by the forward and the reverse sequence primers. An aligned base is considered acceptable if it passes “horizontal scan,” described in the previous section. The numbers of Q20 bases in the total bases and the accepted bases are recorded. The number of bases rejected due to stuttering is also recorded.

In all three datasets, each sample was sequenced in both the forward and the reverse orientations, giving a 2× redundancy in sequence coverage for each sample.

In addition to the read-based coverage described above, we also analyzed the sample-based coverage, which combines the reads from both the forward and the reverse orientations to calculate the total bases and the accepted bases for each sample. At each position in the genomic interval spanned by the forward and the reverse primers, bases from all reads of the same sample are evaluated. An accepted base in one read always overwrites a rejected base in another. For bases with the same status, the higher quality is recorded for the Q20 analysis.

### Run time parameter of Phrap and PolyPhred 5.0.2.

We ran Phred/Phrap/PolyPhred using the template genomic sequences and traces derived from resequencing. Each template sequence was converted into a reference trace using the program SudoPhred. Each base of the reference template sequence was assigned a Phred quality score of 59. Using the default parameters of Phrap, an amplicon with a high SNP rate can be split into multiple contigs. For example, in the mouse resequencing data, 95% of the amplicons were assembled into multiple contigs even after excluding the traces derived from the strain SPRET/Ei (which belongs to the species *M. spretus*). Using the parameters “–repeat_stringency 0.55 –forcelevel 2,” we were able to obtain a single contig in 95% of the amplicons. Therefore, we used this setting in all datasets.

We activated options in PolyPhred to use the template genomic sequence as the reference sequence and to combine forward and reverse reads from the same individual for genotype calls (the “–source” option).

In the results generated by PolyPhred 5.0.2, approximately 50% of the high-quality SNPs (score = 99) were monomorphic in their genotype calls (i.e., PolyPhred's own assessment was monomorphic even though it called a SNP). We visually analyzed 30 such cases, and were able to confirm that they were all monomorphic sites. We developed a filter to remove these monomorphic “SNPs” from the PolyPhred output.

### Analysis of false positive and false negative rates.

We calculated false positive rate to measure the specificity of the three programs tested in this study using the following formula: the number of false positive SNPs divided by the number of SNPs discovered. This formula is referred to as the false discovery rate [23]. We calculated false negative rate to measure sensitivity using the following formula: the number of known missed SNPs divided by the number of all true SNPs.

## Supporting Information

Figure S1 Trace Chromatogram of Four CAST/Ei Animals at *Bach1* Locus (SNP1 in Table 2) All but the second animal (animal B in Table 2) are heterozygous.
(88 KB PDF)

Figure S2 An Example of Discrepancy between Visual Analysis and Genotyping Result The second and the third traces are the reverse and the forward reads from an individual identified as a heterozygote by visual analysis. The minor allele *T* was only found in this individual. The top is a sequence of a homozgygote control. Both SNPdetector and PolyPhred found this SNP (PolyPhred score = 99). However, the genotype result is monomorphic at this site.
(104 KB PDF)

Table S1 SNPdetector Parameters Used to Make Genotype and SNP Calls (113 KB PDF)

## Abbreviations

HQDU: heterozygote Phred quality score drop unit
indel: insertion/deletion
NQS: neighborhood quality standard
SNP: single nucleotide polymorphism
STR: simple tandem repeat
