# Supplementary material for: SNPdetector: A Software Tool for Sensitive and Accurate SNP Detection
Source: PLoS Comput Biol. 2005 Oct 28;1(5):e53. doi: 10.1371/journal.pcbi.0010053 (PMC1274293; doi:10.1371/journal.pcbi.0010053)

Figure s1. Trace chromatogram of four Cast/Ei animals at Bach1 locus (SNP1 in Table 2). All but the second animal (animal B in Table 2) are heterozygous.

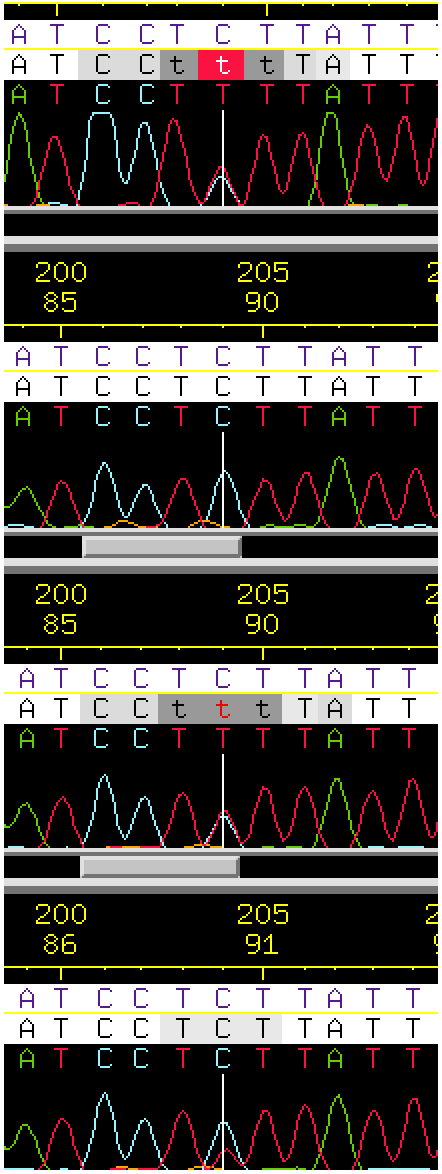

Supplement: Figure S1 — All but the second animal (animal B in Table 2) are heterozygous. (88 KB PDF) [file pcbi.0010053.sg001.pdf]
