# Supplementary material for: SNPdetector: A Software Tool for Sensitive and Accurate SNP Detection
Source: PLoS Comput Biol. 2005 Oct 28;1(5):e53. doi: 10.1371/journal.pcbi.0010053 (PMC1274293; doi:10.1371/journal.pcbi.0010053)

Figure s2. An example of discrepancy between visual analysis and genotyping result. The second and the third traces are the reverse and the forward read from an individual identified as a heterozygote by visual analysis. The minor allele T was only found in this individual. The top is a sequence of a homozgygote control. Both SNPdetector and polyphred found this SNP (polyphred score 99). However, genotype result is monomorphic at this site.

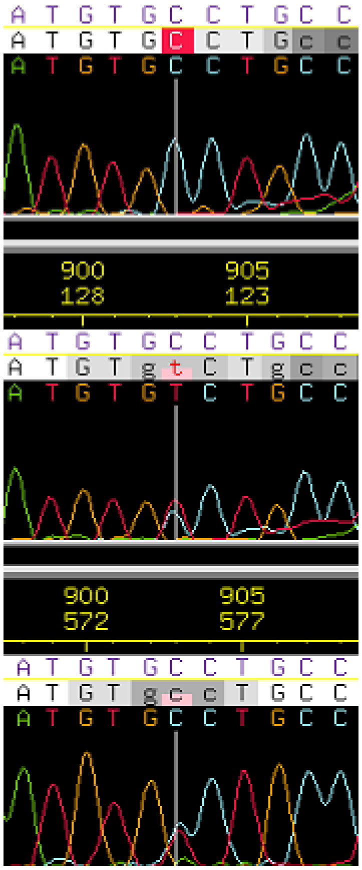

Supplement: Figure S2 — The second and the third traces are the reverse and the forward reads from an individual identified as a heterozygote by visual analysis. The minor allele T was only found in this individual. The top is a sequence of a homozgygote control. Both SNPdetector and PolyPhred found this SNP (PolyPhred score = 99). However, the genotype result is monomorphic at this site. (104 KB PDF) [file pcbi.0010053.sg002.pdf]
