# Supplementary material for: SNPdetector: A Software Tool for Sensitive and Accurate SNP Detection
Source: PLoS Comput Biol. 2005 Oct 28;1(5):e53. doi: 10.1371/journal.pcbi.0010053 (PMC1274293; doi:10.1371/journal.pcbi.0010053)
Supplement: Table S1 — (113 KB PDF) [file pcbi.0010053.st001.pdf]

Table s1. SNPdetector Parameters Used to make Genotype and SNP calls.

Parameter	Type of Variable	Underlying data	Description
**disqualify_by_stutter**	Binary	Ordinal	SNP is within a stutter region
**is_mismatch_cluster**	Binary	Ordinal	4 or more mismatches with phred quality >30
**good_homo_allele**	Binary	Ordinal	Quality check of adjacent bases
**strand_conflict**	Binary	Ordinal	Sample A has only single strand coverage and it has homozygous minor allele. However, in other samples (eg. B or C) with sequences from both orientations, the minor alleles detected in the same direction as sample A were found to be in conflict with the alleles detected in the other orientation. This information was used to evaluate potential sequencing error in those with single-strand coverage.
**pass_dirty_check**	Binary	Continuous	2° peak of het is distinct from "dirty" homo
**neighbor_spill**	Binary	Continuous	The 2° peak of the current position extends to more than 1 base.
**spill_ratio**[a]	Continuous	Continuous	The max 2° peak height divided by the min 2° peak height for a putative "spill" region.
**max_regional_dirty_peaks**[b]	Binary	Continuous	in the surrounding 20bp window, the maximum number of 2° peaks that exceed 80% of the 2° peak height of the current site.
**skip_hetero_analysis**	Binary	Ordinal	>90% of the reads have double peaks and longest region with no 2o peak is less than 50bp
**drop_a1_ratio**[c]	Continuous	Continuous	The peak drop ratio of the first allele in a putative heterozygote compared to its homozygote counter part.
**drop_a2_ratio**	Continuous	Continuous	Same as drop_a1_ratio except comparing the 2° peak to its homozygous read.
**hetero_has_peak_drop**[d]	Categorical	Categorical	**0, no peak drop compared to corresponding homozygotes; 1, heterozygote has a peak drop compared to corresponding homozygotes; 2, fail to find a homozygote with comparable flanking peak profile.**
**flanking_region_score**	Continuous	Continuous	**Maximum of scores determined from flanking 4 bp and flanking 20 bp regions.**
**is_clean_hetero**[e]	Binary	Categorical	**True if 5 tests in footnote e pass.**
**pass_poly_check**[f]	Binary	Categorical	>0 if 2° peaks of a putative SNP site (homozygote or heterozygote) and its 4-bp flanking region have test in footnote e.
**High mismatch** (short range)	Binary	Ordinal	20 bp window <16 are identical to reference
**High mismatch** (long range)	Binary	Ordinal	In a 50 bp window, the distance between short-range High-mismatch segments is <5bp
**Low quality** (short range)	Binary	Ordinal	5 bp window, average quality below 20
**Low quality** (long range)	Binary	Ordinal	In a 50 bp window, the distance between short-range Low-quality segments is <5bp
**Region of 2° peaks**	Binary	Ordinal	10 bp window, 7 positions have 2° peak ratio ≥0.1

(short range)			
**Region of 2° peaks** (long range)	Binary	Ordinal	In a 50 bp window, the distance between shrot-range 2° peak is <5bp
**Region of high 2° pks** (short range)	Binary	Ordinal	10 bp window, 7 positions have 2° peak ratio ≥0.3
**Region of high 2° pks** (long range)	Binary	Ordinal	50 bp window
**Mismatch_cluster** (short range)	Binary	Ordinal	≥2 bp high quality (phred 30) mismatches in a 20 bp window.
**Mismatch_cluster** (long range)	Binary	Ordinal	≥4 bp high quality (phred 30) in a 40 bp window

a, Use of **spill_ratio**. This ratio differentiates a spill from a SNP cluster. The latter tends to have similar secondary peak heights (e.g. spill ratio close to 1) while the former tends to have a large difference.

b, Use of **max_regional_dirty_peaks**. This information was used to determine if the background noise of two traces are comparable in the "vertical" scan.

c, Use of **drop_a1_ratio**. A putative heterozygote site is compared to each of the homozygous read of the same orientation: to determine a) whether the left and the right flanking primary peaks in the two reads are comparable. A –1.0 value is assigned to those with incomparable homozygous flanking peaks; b) else (e.g. the flanking peaks are comparable), normalize the primary peak ratio of the homo/hetero at the SNP site to the average of homo/hetero at the left and the right flanking sites. The average ratio of all pair wise het-to-homo comparison (excluding the –1.0 cases) will be stored.

d, Use of **hetero_has_peak_drop**. This value is initially set by the value of **drop_a1_ratio** of 0.55 (almost 50% reduction of a primary peak) subject to the following revisions:

- The forward and the reverse read have the same genotype and the reduction of the primary peak + the rise of the secondary peak ratio is approximately 1. This shows that the reduction of the primary peak can be explained by the addition of the secondary peak.
- When the secondary peak ratio of a putative heterozygote is less 20% of a dirty homozygote, the peak_drop_ratio is reset to 0.
- If a heterozygote has clean flanking region and its reduction of the primary peak can be explained by the addition of the secondary peak, then the flag is set to 1.

A non-clean heterozygote is used for SNP call only when its **hetero_has_peak_drop** flag was set to 1.

e, Determination of **is_clean_hetero**.

i. The putative heterozygote does not fit into a "spill" profile, i.e. a neighboring homozygote followed by at least 2 secondary peaks (with diminishing secondary peak area ratio) in its neighbor. This profile is evaluated with a sliding window method.

ii. The heterozygote does not have any indel on its immediate left or right side.

iii. The secondary peak represents a residue different from those of the primary peaks of its left and right neighbors.

iv. There are no drastic peak height differences between the primary peak of the putative heterozygote site and its left/right neighbors. Specifically, the primary peak height should be ≥1/6 of its neighbor and ≤2 of its neighbor. If both the left and the right neighbor fail to meet this criterion, then the site fails in this test. The ≥1/6 test ensures that the site does not look like a deep valley (normally indicates a potential sequencing error). The ≤2 test will exclude a site if the primary peak appears to be twice as high as its neighbor because a heterozygote is expected to have its primary peak reduced compared to a homozygote. The reduced primary peak usually has lower peak height than its neighbors.

v. The flanking region, excluding those that may appear to be putative heterozygote (secondary peak ratio ≥0.70), contains no site of secondary peak ratio ≥5%. If the secondary peak ratio of a putative heterozygote is below 60, then the test requires absence of secondary peak in the flanking region.

“#” in output indicates that a putative heterozygote has no noisy background (i.e. is**_clean_hetero**) nor apparent abnormalities in both its primary and secondary peaks compared to its immediate neighbors.

f, Calculation of **pass_poly_check**:. Define P= (secondary_peak_area/primary_peak_area)*100 (i.e. percent of primary peak area occupied by secondary peak). To evaluate noise at the flanking regions of a putative heterozygote or a homozygote, the program checks the secondary peak of each base in the flanking region. If each base in the flanking region passes the test of (P ≤ 0, ≤ 10, ≤ 20), then the flanking region is considered to have no, little, limited noise. To avoid penalizing secondary peak of a potential heterozygote in the flanking region, a site with a secondary peak ratio ≥ 0.70 is skipped. At the SNP site, the same test is applied to measure the noise level at a homozygous genotype. For a heterozygous genotype, the high, med and low is rewarded to those with P ≥ 80, ≥ 50 and ≥ 35 respectively. $ in output indicates pass_poly_check is greater than 0.
